# Association of Rural Hospital Admissions with Access, Treatment, and Mortality for Patients with Acute Myocardial Infarction in Shanxi, China

**DOI:** 10.3390/ijerph19116382

**Published:** 2022-05-24

**Authors:** Ding Tao, Ya Sun

**Affiliations:** 1 School of Data Science, The Chinese University of Hong Kong, Shenzhen 518172, China; dingtao@link.cuhk.edu.cn; 2 School of Economics, Huazhong University of Science and Technology, Wuhan 430074, China

**Keywords:** instrumental variables, acute myocardial infarction, rural China, quality of care

## Abstract

China recently launched healthcare reforms to reduce disparities in healthcare resources between urban and rural areas. However, few studies have determined how admission to rural hospitals has affected patient care and outcomes. This study aims to determine whether admission to a rural hospital is associated with changes in treatment and outcomes. Using a province-wide, administrative database of 62,380 patients (51,355 urban patients vs. 11,025 rural patients) with acute myocardial infarction (AMI) in Shanxi from 2015 to 2017, we identified the differential distance from the patient’s residential address to the nearest hospital and the nearest percutaneous coronary intervention (PCI)-capable hospital as instrumental variables. We estimated the risk-adjusted differences in outcomes and treatments for patients admitted to rural hospitals versus urban hospitals using a two-stage least squares instrumental variable analysis method. Based on instrumental variable analysis, admission to a rural hospital was associated with a 5.3% (95% CI, 0.012 to 0.093; *p* = 0.011) increase in mortality. There was a 59.8% (95% CI, −0.733 to −0.463; *p*-values < 0.0001) decrease in receiving PCI, an 18.8% (95% CI, −0.231 to −0.146; *p*-values < 0.0001) decrease in receiving fibrinolysis, and a 71.8% (95% CI, 0.586 to 0.849; *p*-values < 0.0001) increase in receiving medication-only treatment for patients admitted to rural hospitals. Rural hospitals in China thus offer relatively poor care for myocardial infarction. Hospital facilities and reperfusion therapies must be improved.

## 1. Introduction

Healthcare disparities between urban and rural areas have attracted increasing attention around the world [1,2,3,4,5]. Studies based on US contexts have shown that patients with acute myocardial infarction (AMI) have higher mortality rates in rural hospitals than in urban hospitals [1,2,3]. Similar results were also found in South Korea [4]. While urban–rural differences in treatment and outcomes for patients with AMI are well studied in the United States, much larger gaps in knowledge exist in other countries, especially in low- and middle-income countries (LMIC).

Shanxi Provincse in China represents prominent features of a typical LMIC with an average gross domestic product per capita slightly above 7000 USD, similar to the level of Thailand [6]. As with many LMICs, rural hospitals in Shanxi are typically primary or secondary hospitals, have lower volumes of AMI patients, have limited specialist care, and lack PCI capability [7]. As the government of China has endorsed the improvement of specialist care in rural hospitals, particularly for cardiac care, since 2014, the number of rural hospitals capable of PCI has increased over the past five years [8]. Previous studies have shown that gaps between urban-rural differences in evidence-based treatments have been decreased in China [7,9,10]. However, there is limited information on the association between admission to rural hospitals and outcomes. Given the similarities between Shanxi and LMICs, studying the associations of rural hospital admission and outcomes in Shanxi could contribute by identifying different patterns than those observed in the United States and provide insight for LMIC countries of a similar economic level.

This study determines the extent to which admission to a rural hospital has been associated with access to specialist care, treatment, and health outcomes for patients with AMI. Using an instrumental variable (IV) approach, we hypothesized that admission to rural hospitals is associated with worse quality of care for patients with AMI through a lower likelihood of receiving reperfusion and increased mortality.

## 2. Materials and Methods

### 2.1. Data Transparency

Due to the sensitive nature of the data, requests to access the dataset may be sent to the Health and Family Planning Commission of Shanxi, China.

### 2.2. Data Sources

The primary source of individual-level data was electronic medical record data from patients hospitalized between 2013 and 2017 in Shanxi, China, provided by the Health and Family Planning Commission of Shanxi Province. The dataset includes information on patient demographic characteristics (age, sex, race, marital status, and job status), length of stay, up to 10 secondary diagnoses coded using the International Classification of Disease, Tenth Revision (ICD-10), up to seven procedures coded using the International Classification of Disease, Ninth Revision (ICD-9), severity of disease (normal, severe, or dangerous), inpatient mortality, and medical spending. Unique identifiers at the patient level, such as names and identity card numbers, were excluded before the authors were granted access. Huazhong University of Science and Technology’s institutional review board reviewed the study protocol and waived the requirement for participants’ informed consent owing to the infeasibility of acquiring consent for medical record data.

### 2.3. Patient Selection

**Figure 1:**
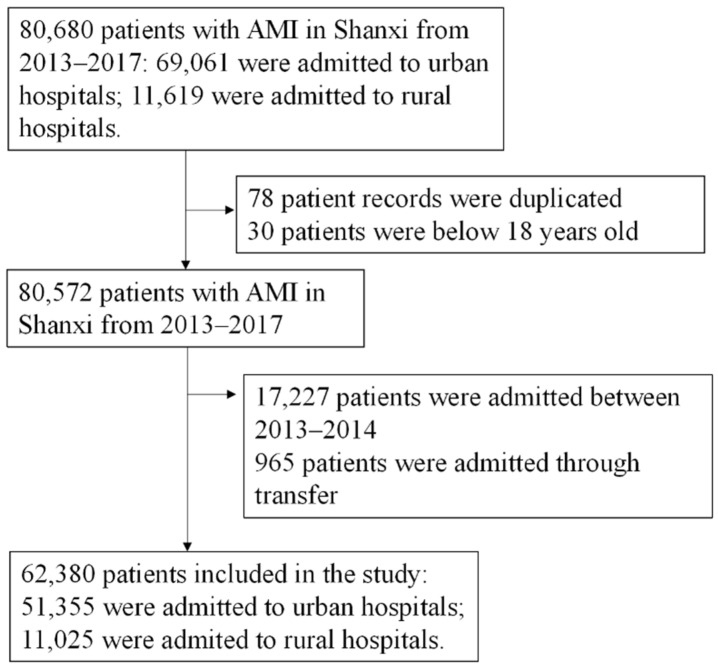
Flow chart of patient selection.

Following previous literature, patients with AMI were identified by ICD-10 codes (ICD-10 codes: I21) from both the inpatient discharge database and the emergency department discharge database. The patient universe began with 80,680 cases of AMI between 2013–2017 (Figure 1). Based on case information, 78 patient records were duplicated, and the duplications were removed. Thirty patients with AMI were under 18 years of age and thus excluded from the analysis as AMI under 18 was rare. As we are interested in the associations between admission to a rural hospital and outcomes after the government’s endorsement in 2014, we only retained patient records from 2015–2017, removing 17,227 patient cases. To evaluate the effect of initial admission, we excluded 965 patients who were transferred from other hospitals. Thus, our analytical sample consisted of 62,380 patients.

### 2.4. Patient and Public Involvement

This research was conducted without patient involvement.

### 2.5. Outcomes

The prespecified primary outcomes relevant to associations between admissions to rural hospitals included (1) risk-adjusted inpatient mortality, (2) inpatient length of stay, (3) 30-day cardiac readmission, and (4) inpatient spending, including both total and out-of-pocket expenditure.

The secondary outcomes included the receipt of (1) PCI treatment, (2) fibrinolytics, (3) medication alone, and (4) coronary angiography.

As primary outcomes, inpatient mortality and 30-day risk-standardized readmission rates were used, as previous studies have indicated that intensity of care for AMI patients has short- and long-term effects. Inpatient mortality and length of stay capture the association between admission to a rural hospital and quality of care. Total inpatient spending and out-of-pocket expenditure were also examined.

The secondary outcomes captured procedures received by each patient with AMI. In particular, we used the following ICD-9 procedures codes: PCI (00.66, 36.00–36.09) and fibrinolytic therapy (99.10) during the hospitalization. We defined the patient as having received only medication treatment if surgical records were blank or filled out as “null” and medical expenditure was positive. Medication-only treatment was included because any increases in specialist care would lead to decreases in medication-only treatments. We included coronary angiography (ICD-9 procedure codes 88.50–88.57 to capture any attempts at intervention.

### 2.6. Rural and Urban Classifications

Hospitals were classified as rural or urban based on whether the hospital was located in a metropolitan statistical area. If the hospitals were located in a “Shi” (city) or “Qu” (district), they were classified as urban hospitals. Hospitals that were located in a “Xian” (county) or “Xiang” (village) were classified as rural hospitals.

### 2.7. Statistical Analysis

The criterion-standard approach to answering the question uses randomized control trials, which require patients to be randomly assigned to a rural or urban hospital. Although such a trial would promise causal interpretation, it would generally be too costly, too difficult, or unethical to perform. Consequently, the majority of previous studies on urban-rural disparities in China use observational data, which could potentially suffer from confounding factors such as selection bias [7,8,9].

To enhance causal identification of the effect from admissions to rural hospitals, we used the IV method with the differential distance from the patient’s residential address to the nearest hospital and the nearest PCI-capable hospital as instruments. Patients often seek treatment at hospitals that are near their homes; thus, patients’ residences should be highly predictive in determining the type of initial hospital admission. However, it is unlikely that patients’ residences would influence patient outcomes directly. By employing the differential distances as instruments, we essentially reallocate patients into groups that are identical to each other except for their IV values. We are thus able to isolate the effect of treatment (i.e., admission to a rural hospital) in the observational data that is independent of unobserved patient characteristics (i.e., patient preference and severity of comorbidities) [11,12,13,14].

We first compared outcomes via multivariable adjusted ordinary least squares (OLS) linear regression to motivate the IV approach. Covariates included demographic characteristics (i.e., gender, profession, and marital status) and existence of comorbidities (i.e., stroke, diabetes, and hypertension). We then performed the adjusted IV analyses using the two-stage least squares methodology. In the first stage, we built a linear regression model predicting admission to rural hospitals with the instrument. In the second stage, we employed a least square estimation with the predicted values from stage one as the main predictor. The same covariates were used for the IV approach. Associations between admissions to rural hospitals and outcomes were estimated based on the coefficient of the IV in the stage 2 model. Robust standard errors were estimated for all analyses.

The medical record system requires patients to provide address information upon admission, including residential, work, and birthplace addresses. Our primary information source for personal addresses was the residential address. If it was missing, we replaced it with a work address or, if the work address information was also missing, with a birthplace address. In total, the residential address information was missing for 1572 individuals and required replacements.

We used the built-in Tencent Map function to identify the nearest hospital of any type to the patient’s residential address. The Tencent Maps service was also used to calculate the differential distance, actual driving distance, and distance to the next hospital.

### 2.8. Evaluation of IV Assumptions

The instrument’s validity depends on three assumptions [14,15]. First, the instrument should be correlated with treatment choice. In the first stage, we showed that distance has a strong relationship with the type of hospital to which a patient with AMI is initially admitted. We evaluated the strength of this assumption through F-statistics from the first stage. Second, the exclusion restriction assumption requires that the instrument affects patients’ outcomes (e.g., mortality) only through the hospital choice. This assumption cannot be empirically verified; however, it seems reasonable to assume that patients do not choose their residential address based on the severity of their diseases [12]. Nonetheless, we performed falsification tests by restricting the sample to patients whose government registration address was the same as their current residential address to provide suggestive evidence. Third, the IV should effectively randomize patients so that patients should be similar in measured and unmeasured characteristics. We compared patient characteristics using the median distance (three-minute driving distance) as the cut-off point to provide suggestive evidence.

We report first-stage results using the differential distance both as a single scalar IV and a more flexible specification to account for the variation in the distance measure. Patients were stratified into five groups according to the quintiles of differential distance distribution. The fifth quintile group (more than 17 min) was the base group. The coefficients show that the shorter the differential distance, the lower the chance of being admitted to a rural hospital. Our second-stage results are based on this specification.

## 3. Results

### 3.1. Patient Characteristics

Table 1 presents the patient characteristics of our sample. Out of 62,380 total patients in this study, 11,025 patients were admitted to rural hospitals, and the inpatient volume of urban hospitals was more than four times that of rural hospitals. Patients admitted to rural hospitals were older, more likely to be female, and much more likely to be farmers than those admitted to urban hospitals. With the national health insurance reform that provides universal coverage, patients with the New Cooperative Medical Scheme, the insurance designed for rural citizens, were more likely to be admitted to rural hospitals (69.50% vs. 44.20%). In terms of comorbidities, patients admitted to rural hospitals were less likely to have diabetes, hypertension, and stroke than those admitted to urban hospitals. In addition, individuals admitted to rural hospitals were less likely to be STEMI patients and more likely to be NSTEMI patients than those admitted to urban hospitals.

### 3.2. OLS Comparison

The in-hospital mortality rates and 30-day readmission rates are statistically no different between patients admitted to rural hospitals and those admitted to urban hospitals under the OLS estimation (Table 2). However, patients admitted to rural hospitals have a shorter length of stay (−1.601, 95% CI, −1.74 to −1.46), lower total inpatient spending (−15,985, 95% CI, −16,520 to −15,451), and lower out-of-pocket spending (−6882, 95% CI, −7226 to −6538). These results are consistent with previous studies on China’s rural and urban healthcare disparities [7,9]. If these results were unbiased, they would indicate that rural hospitals in China offer not only better but also more effective care for patients with AMI, as suggested by Li et al. [9].

### 3.3. IV Comparison

The IV estimates show that admission to a rural hospital for AMI patients is associated with 5.3% higher mortality (95% CI, 0.012 to 0.093; Table 2). This estimate is substantially different from those estimated using the OLS analytical approach. Initial admission to a rural hospital diminishes the survival chances of the patient significantly, suggesting worse quality of care in rural hospitals for patients with AMI. In terms of treatment procedures, patients admitted to rural hospitals are 59.8% less likely to receive PCI (95% CI, −0.733 to −0.463) and 18.8% less likely to receive fibrinolysis (95% CI, −0.231 to −0.146) but are 71.8% more likely to receive medication-only treatment (95% CI, 0.586 to 0.849). Although admission to a rural hospital is also associated with a lower rate of coronary angiography, the magnitude is much smaller than PCI (35.6% vs. 59.8%). Since coronary angiography is an essential procedure in conducting PCI, the result suggested that many patients admitted to rural hospitals may undergo coronary angiography but are not ultimately given PCI.

We conducted sensitivity analysis by examining subgroups of the patient population according to profession and insurance type (Supplementary Tables S1 and S2). The results show that farmers are more likely to be affected by being initially admitted to rural hospitals. On average, admission to rural hospitals leads to a 3.7% higher mortality rate for farmers but has no significant effects for non-farmers. The estimation results indicate that rural populations in China are suffering from a lack of quality care in rural hospitals. Urban populations are largely spared this effect, as the majority live near urban hospitals.

We also investigate the sensitivity of the results by patients’ main diagnosis (Supplementary Table S3). Results for patients with STEMI and Non-STEMI were reported. The results show that patients whose main diagnosis was non-ST-elevation myocardial infarction (STEMI) were more likely to be affected from being initially admitted to rural hospitals. Being admitted to rural hospitals leads to 4.45% higher mortality rate for patients with non-STEMI but has no significant effects for patients with STEMI. For patients whose main diagnosis was STEMI, being admitted to rural hospitals was associated with significantly lower PCI rates (68.3%) but no differences in receiving fibrinolysis. Though we observe no significant differences in mortality for patients with STEMI, being admitted to rural hospitals leads to 1.9% higher 30-day readmission rates, suggesting worse quality of care.

In Supplementary Table S4, we check the differences between rural and urban hospitals in number of tests performed if PCI was conducted. We observe no differences in number of X-rays and electrocardiography (ECG) performed between rural and urban hospitals.

### 3.4. Evaluation of the Instrument

The first-stage regression demonstrates a Cragg-Donald Wald F statistic of 146.4, much higher than the generally accepted threshold of 10, suggesting strong instruments that are highly predictive of actual rural hospital admission (Supplementary Table S5). To demonstrate the reduced differences in individual characteristics using the IV method, we separated the patients with AMI into two groups based on differential distance using the median distance (three-minute driving distance) as the cut-off point (Table 3). This practice helped show not only the relevance of the IVs with the variable of interest (in this case, the rurality of the hospital) but also the balanced patient characteristics based on IVs. Compared with the data presented in Table 1, the differences among the groups become smaller in all characteristics, as compared to a direct comparison of urban and rural hospitals. This comparison provides some validation for the key assumption of the IV technique: The distribution of unobserved characteristics is balanced across differential distances.

## 4. Discussion

We applied the IV method to estimate the association between rural hospital admission and outcomes for patients with AMI in Shanxi, China. The descriptive statistics are in line with earlier studies that showed patients with AMI who were admitted to rural hospitals differed substantially in demographic characteristics and had fewer comorbidities in general [7,9]. The estimates obtained using OLS linear regressions are consistent with those of previous studies and indicate no significant difference between urban and rural hospital quality of care. Our IV results, however, indicate that the estimates from these models suffer from selection bias because hospital choice at admission is likely to be confounded by unobserved patient characteristics.

The results from the IV method show that admission to rural hospitals is associated with a significantly higher likelihood of in-hospital mortality. With respect to treating patients with AMI, the proportion of rural hospitals offering PCI is significantly lower than that of urban hospitals. IV estimation according to profession shows that farmers are more likely to receive inferior quality of care in rural hospitals, suggesting that rural residents are the group suffering the most from admission to rural hospitals.

Although the findings are consistent with previous studies on the Chinese healthcare system showing that county-level hospitals have worse health facilities and lower reperfusion rates, our identification strategy reveals the correlation of admission to a rural hospital rather than medical procedures per se [8,16]. In other words, the higher mortality rate in rural hospitals is a result of not only a lack of PCI and fibrinolysis treatments but also a lack of skilled hospital staff or specialists. Our findings are also in line with earlier findings, which have shown that patients with AMI benefit from invasive procedures. For example, Keeley et al. [17] found that percutaneous transluminal coronary angioplasty is better than thrombolytic therapy at reducing short-term mortality risk. Studies with observational data using similar IV designs also showed the benefits of invasive procedures, though mostly short-term [12,14,18].

Our results contribute to the literature in three ways. First, to our knowledge, this is the first study to apply the IV method to estimate the association between rural hospital admission and outcomes for patients with AMI in China. Second, our findings are particularly relevant from a health-provision perspective. The results could help policymakers optimize the allocation of medical resources and hospital specializations between rural and urban regions. In particular, we have two recommendations. One is that we would recommend improving quality of care by investing more both in the medical infrastructure and training of human resources of rural hospitals. Access to cardiac catheterization facilities and specialized services is required for procedural management of AMI, which is typically unavailable in rural hospitals. As rural hospitals are usually short of specialists, training the ability of physicians in rural hospitals to diagnose and treat AMI at an early stage could effectively reduce the mortality rates at rural facilities. The other is to increase the quality of diagnosis and treatment, telehealth and mHealth could be employed in rural hospitals. Telehealth has proven to be beneficial for rural practitioners dealing with an emergency, and it can include remote specialist interpretation of an ECG tracing, remote prescribing, and therapeutic training and monitoring. Third, our findings raise the importance of including rural hospitals in clinical research. Our findings highlight the substantial differences between rural and urban hospitals in patient composition, medical practices and quality of care. Including rural population in clinical trials could significantly improve outcomes, quality of life and equity of access in health care.

Our study has some limitations. First, we only considered patients who survived until hospital admission. Individuals who died en route to the hospital could potentially have influenced our results. Second, because we did not have data on mortality after discharge, we could not provide evidence on the medium- and long-term effects of being admitted to a rural hospital. Future studies should observe the differences in mortality between patients admitted to rural and urban hospitals after discharge, as the urban-rural disparity in quality of care could lead to worse medium- and long-term survival outcomes among patients. Third, we did not have sufficient information identifying the clinical type of AMI [19]. As patients admitted to rural hospitals were older, more likely to be female, we speculate that a higher percentage of patients were Type 2 AMI in rural hospitals [19,20]. Since indications to PCI and fibrinolysis were different according to type of MI, percentage of type 2 patients could potentially influence our results.

## 5. Conclusions

This study shows that rural hospitals illustrate inferior quality of care for patients with AMI than urban hospitals in China. Our findings indicate that an initial admission to a rural hospital may affect the survival chances of patients with AMI significantly and negatively. To achieve better quality and equitable care for patients with AMI, more intensive investigations into the underlying mechanisms of physician treatment decisions in rural hospitals and targeted efforts to reinforce treatment standards are essential. Given the trend of rapid aging and the vast population residing in rural regions, this mission is both urgent and essential for China’s policymakers.

## Figures and Tables

**Table 1:** Baseline patient characteristics.

Characteristics	Admitted to Urban Hospitals (*n* = 51,355)	Admitted to Rural Hospitals (*n* = 11,025)	*p* Value
Age, y (SD)	61.82 (12.76)	63.08 (12.62)	<0.0001
Female, no. (%)	12,869 (25.1)	3037 (27.93)	<0.0001
Farmers, no. (%)	26,013 (50.65)	8924 (80.94)	<0.0001
Insurance, no. (%)			
UEBMI	17,631 (34.33)	1542 (13.99)	<0.0001
URBMI	3561 (6.93)	456 (4.14)	<0.0001
NCMS	22,700 (44.20)	7662 (69.50)	<0.0001
Other insurance	1445 (2.81)	71 (0.64)	0.0002
No insurance	5034 (9.80)	954 (8.65)	<0.0001
Diabetes, no. (%)	11,641 (22.67)	1775 (16.10)	<0.0001
Hypertension, no. (%)	25,332 (49.33)	4789 (43.44)	<0.0001
Stroke, no (%)	6115 (11.91)	869 (7.88)	<0.0001
STEMI	8308 (16.18)	648 (5.88)	<0.0001
NSTEMI	43,047 (83.82)	10,377 (94.12)	<0.0001
**Primary Outcomes**			
Mortality rate (SD)	0.021 (0.14)	0.021 (0.14)	0.541
Length of stay (SD)	11.10 (6.35)	8.95 (7.38)	<0.0001
30-day readmission rate (SD)	0.005 (0.07)	0.005 (0.07)	0.865
Inpatient spending (SD)			<0.0001
Total expenditure	32,397.12 (26,093.17)	13,365.53 (16,056.45)	<0.0001
Out-of-pocket expenditure	7628.12 (17,536.25)	575.62 (2454.21)	<0.0001
**Secondary Outcomes, no. (%)**			
PCI rate	23,275 (45.32)	1203 (10.91)	<0.0001
Fibrinolysis	1099 (2.14)	23 (0.21)	<0.0001
Coronary angiography	25,306 (49.28)	1348 (12.23)	<0.0001
Medication only	19,694 (38.35)	9364 (84.93)	<0.0001

Note: NCMS: New Cooperative Medical Scheme; PCI: percutaneous coronary intervention; SD: standard deviation; UEBMI: Urban Employee Basic Medical Insurance; URBMI: Urban Resident Basic Medical Insurance. STEMI: ST elevation myocardial infarction. NSTEMI: Non-ST elevation myocardial infarction.

**Table 2:** Primary results—OLS and IV analysis.

	OLS			IV		
Primary Outcomes	Being Admitted to Rural Hospitals	SE	*p* Value	Being Admitted to Rural Hospitals	SE	*p* Value
Mortality	0.002	0.002	0.346	0.053	0.021	0.011
Length of stay	−1.606	0.074	<0.0001	−6.436	0.994	<0.0001
30-day cardiac readmission	−0.001	0.001	0.107	0.003	0.01	0.79
Total inpatient spending	−15,985.7	272.6	<0.0001	−44,846	3870	<0.0001
Out-of-pocket spending	−6882.6	175.7	<0.0001	−46,839	3115	<0.0001
**Secondary Outcomes**						
PCI rate	−0.316	0.005	<0.0001	−0.598	0.069	<0.0001
Fibrinolysis	−0.018	0.002	<0.0001	−0.188	0.022	<0.0001
Coronary Angiography	−0.344	0.005	<0.0001	−0.357	0.067	<0.0001
Medication only	0.43	0.005	<0.0001	0.718	0.067	<0.0001

Note: All regressions controlled for gender, age, race, marital status, profession, severity at admission, insurance status, comorbidities, and year fixed effects. The instrumental variable (IV) analysis reports the second-stage results. OLS: ordinary least squares; PCI: percutaneous coronary intervention; SE: standard error.

**Table 3:** Baseline patient characteristics across levels of the instrumental variable.

Characteristics	Admitted to Hospitals Below Median Distance (*n* = 31,191)	Admitted to Hospitals Above Median Distance (*n* = 31,189)	*p* Value
Age, y (SD)	62.02 (12.8)	62.07 (12.6)	0.059
Female, no. (%)	7761 (24.9)	8145 (26.2)	0.0003
Farmers, no. (%)	14,510 (46.5)	20,427 (65.5)	<0.0001
Insurance, no. (%)			
UREMI	11,021 (35.33)	8152 (26.1)	<0.0001
URBMI	2434 (7.8)	1583 (5.1)	<0.0001
NCMS	12,124 (38.9)	18,238 (58.5)	<0.0001
Other insurance	1081 (3.47)	435 (1.39)	<0.0001
No insurance	3767 (12.08)	2221 (7.12)	<0.0001
Diabetes, no. (%)	7208 (23.1)	6208 (19.9)	<0.0001
Hypertension, no. (%)	15,168 (48.6)	14,953 (47.9)	0.09
Stroke, no (%)	3541 (11.4)	3443 (11.0)	0.21
STEMI	5444 (17.45)	3512 (11.26)	<0.0001
NSTEMI	25,747 (82.55)	27,677 (88.74)	<0.0001
**Primary Outcomes**			
Mortality rate (SD)	0.019 (0.14)	0.022 (0.15)	0.032
Length of stay (SD)	10.9 (6.77)	10.47 (6.41)	<0.0001
30-day readmission rate (SD)	0.005 (0.07)	0.005 (0.07)	0.57
Inpatient spending (SD)			
Total expenditure	30,771.6 (26,924.9)	27,285.3 (24,219.5)	<0.0001
Out-of-pocket expenditure	7849.4 (18,027.6)	4913.8 (13,915.5)	<0.0001
**Secondary Outcomes, no. (%)**			
PCI rate	12,704 (0.40)	11,774 (0.38)	<0.0001
Fibrinolysis	736 (2.36)	386 (1.24)	<0.0001
Coronary angiography	13,548 (43.44)	13,106 (42.02)	<0.0001
Medication only	13,902 (44.57)	15,156 (48.59)	<0.0001

Note: NCMS: New Cooperative Medical Scheme; PCI: percutaneous coronary intervention; SD: standard deviation; UEBMI: Urban Employee Basic Medical Insurance; URBMI: Urban Resident Basic Medical Insurance. STEMI: ST elevation myocardial infarction. NSTEMI: Non-ST elevation myocardial infarction.

## Data Availability

Requests to access the dataset may be sent to the Health and Family Planning Commission of Shanxi, China.

## Supplementary Materials

The following supporting information can be downloaded at: https://www.mdpi.com/article/10.3390/ijerph19116382/s1, Table S1: Sensitivity Analysis by Profession; Table S2: Sensitivity Analysis by Insurance Type; Table S3: Sensitivity Analysis by Main Diagnosis; Table S4: Differences in Number of Tests between Rural and Urban Hospitals; Table S5: Instrumental Variables’ First-Stage Results..
