# Supplementary material for: Association of Rural Hospital Admissions with Access, Treatment, and Mortality for Patients with Acute Myocardial Infarction in Shanxi, China
Source: Int J Environ Res Public Health. 2022 May 24;19(11):6382. doi: 10.3390/ijerph19116382 (PMC9180441; doi:10.3390/ijerph19116382)
Supplement: Supplementary file 1 [file ijerph-19-06382-s001.zip › ijerph-1702023-supplementary.pdf]

Table S1. Sensitivity Analysis by Profession.

	Farmers			Non-farmers		
**Primary outcomes**	**Admission to rural hospitals**	**SE**	**P value**	**Admission to rural hospitals**	**SE**	**P value**
Mortality	0.037	0.022	0.087	-0.018	0.027	0.496
Length of stay	-6.164	1.114	<0.0001	-7.249	1.297	<0.0001
30-day cardiac readmission	0.018	0.015	0.242	-0.0005	0.009	0.959
Total inpatient spending	-42,604.8	4,432.7	<0.0001	-47,381	4,924.5	<0.0001
Out-of-pocket spending	-48,604.9	4,156.8	<0.0001	-28,547.1	3,014.8	<0.0001
**Secondary outcomes**						
PCI rate	-0.589	0.084	<0.0001	-0.63	0.084	<0.0001
Fibrinolysis	-0.225	0.03	<0.0001	-0.066	0.023	<0.0001
Coronary angiography	-0.489	0.082	<0.0001	-0.329	0.082	<0.0001
Medication only	0.771	0.085	<0.0001	0.705	0.08	<0.0001

All regressions controlled for gender, age, race, marital status, profession, severity at admission, insurance status, co-morbidities, and year fixed effects. The instrumental variable analysis reports the second-stage results. PCI: percutaneous coronary intervention; SE: standard error.

Table S2. Sensitivity Analysis by Insurance Type.

	Farmers			Non-farmers		
**Primary outcomes**	**Admission to rural hospitals**	**SE**	**P value**	**Admission to rural hospitals**	**SE**	**P value**
Mortality	0.039	0.018	0.026	0.002	0.027	0.935
Length of stay	-4.02	0.9	<0.0001	-8.19	1.22	<0.0001
30-day cardiac readmission	0.014	0.013	0.276	-0.0002	0.009	0.976
Total inpatient spending	-45,603.9	3,857.55	<0.0001	-43650.6	4,558.1	<0.0001
Out-of-pocket spending	-50,152.8	3,666.5	<0.0001	-22,068.2	2,726.8	<0.0001
**Secondary outcomes**						
PCI rate	-0.6001	0.08	<0.0001	-0.498	0.102	<0.0001
Fibrinolysis	-0.212	0.028	<0.0001	-0.088	0.028	<0.0001
Coronary angiography	-0.395	0.078	<0.0001	-0.295	0.1007	<0.0001
Medication only	0.776	0.08	<0.0001	0.427	0.098	<0.0001

All regressions controlled for gender, age, race, marital status, profession, severity at admission, insurance status, co-morbidities, and year fixed effects. The instrumental variable analysis reports the second-stage results. PCI: percutaneous coronary intervention; SE: standard error.

Table S3. Sensitivity Analysis by Main Diagnosis

	STEMI			Non-STEMI		
**Primary outcomes**	**Admission to rural hospitals**	**SE**	**P value**	**Admission to rural hospitals**	**SE**	**P value**
Mortality	0.0465	0.037	0.205	0.042	0.022	0.062
Length of stay	-8.788	1.762	<0.0001	-4.188	1.039	0.000
30-day cardiac readmission	0.019	0.014	0.182	0.003	0.012	0.776
Total inpatient spending	-50,358.3	7617.93	<0.0001	-30809.57	3783.13	<0.0001
Out-of-pocket spending	-41,694.3	5989.55	<0.0001	-35214.39	2855.63	<0.0001
**Secondary outcomes**						
PCI rate	-0.627	0.126	<0.0001	-0.356	0.072	<0.0001
Fibrinolysis	0.021	0.017	0.230	-0.209	0.025	<0.0001
Coronary angiography	-0.108	0.120	0.370	-0.298	0.072	<0.0001
Medication only	0.708	0.116	<0.0001	0.485	0.070	<0.0001

All regressions controlled for gender, age, race, marital status, profession, severity at admission, insurance status, co-morbidities, and year fixed effects. The instrumental variable analysis reports the second-stage results. PCI: percutaneous coronary intervention; SE: standard error.

Table S4. Differences in Number of Tests between Rural and Urban Hospitals

	PCI		
**Primary outcomes**	**Admission to rural hospitals**	**SE**	**P value**
Number of X-rays	-0.030	0.021	0.149
Number of ECG	-0.009	0.021	0.658

All regressions controlled for gender, age, race, marital status, profession, severity at admission, insurance status, co-morbidities, and year fixed effects. The instrumental variable analysis reports the second-stage results. PCI: percutaneous coronary intervention; ECG: electrocardiography; SE: standard error.

Table S5. Instrumental Variables' First-Stage Results.

	**Admission to a rural hospital**	**SE**	**P value**	**Admission to a rural hospital**	**SE**	**P value**
Differential distance	0.002	0.0001	<0.0001		0.0001	<0.0001
0–1 min				-0.087	0.005	<0.0001
1–3 min				-0.051	0.005	<0.0001
3–7 min				-0.058	0.005	<0.0001
7–17 min				-0.047	0.005	<0.0001
N		60,096			60,096	
Adjusted R-squared		0.093			0.095	
F-statistic		154.29			146.42	

SE: standard error.
